# Photopolymerization-Induced Phase Separation in a Polypropylene Glycol-Modified Dimethacrylate System

**DOI:** 10.3390/polym18182251

**Published:** 2026-09-15

**Authors:** Irina D. Shapiro, Irina O. Plyusnina, Nikita Yu. Budylin, Uliana V. Nikulova, Aleksey V. Shapagin

**Affiliations:** Frumkin Institute of Physical Chemistry and Electrochemistry, Russian Academy of Sciences (IPCE RAS), 31-4, Leninsky Prospect, 119071 Moscow, Russia; animashir@yandex.ru (I.D.S.); irinaplyusninar@yandex.ru (I.O.P.); budylin_nikita@mail.ru (N.Y.B.); ulianan@rambler.ru (U.V.N.)

**Keywords:** dimethacrylate, polypropylene glycol, UV curing, phase separation

## Abstract

The effect of polypropylene glycol (PPG) on the phase structure formation and deformation properties of a photocurable system based on bisphenol-A 10-ethoxylate dimethacrylate (BPADMA) was studied. Optical interferometry established the complete compatibility of the components before polymerization, and the critical PPG concentration predicted as corresponding to the center of the phase inversion region (28 vol.%) was determined. SEM confirmed the formation of the predicted phase structure, characterized by interpenetrating continuous phases. FTIR spectroscopy showed that the introduction of PPG has virtually no effect on the conversion degree (92.3% for neat BPADMA and 91.5% for BPADMA–PPG blend prepared by volume). The enhanced deformation capacity and reduced compressive resistance are attributed to the formed phase structure.

## 1. Introduction

Photocurable dimethacrylate systems are widely used in the development of functional polymer materials, including protective coatings, adhesives, 3D printing materials, and dental composites [1,2,3]. The widespread application of these systems is due to their high photocuring rate, their ability to undergo polymerization at room temperature, and the elimination of volatile solvent emissions [4]. Despite these advantages, the high cross-linking density of cured dimethacrylate networks often leads to increased brittleness, which limits their use in dental materials and additive manufacturing. One approach to regulating the mechanical properties of such systems involves the introduction of a modifier capable of altering the polymer network structure [5] and inducing a phase-separated morphology.

To regulate the structure and properties of dimethacrylate systems, they are often modified with thermoplastics [6]. The introduction of a thermoplastic modifier soluble in the initial monomer system is accompanied by reaction-induced phase separation during photocuring. The process of structure formation during polymerization in a dimethacrylate–thermoplastic mixture is complex, since an increase in viscosity in the system with an increase in the conversion degree is accompanied by a deterioration in the thermodynamic compatibility of the components, which in most cases leads to phase separation [7,8,9]. Consequently, depending on the choice of the component ratio and the curing temperature, various types of phase structure can form on the temperature–concentration field of the phase diagram—matrix-dispersion, inverted matrix-dispersion, and an interpenetrating phase (IPP) structure—which makes it possible to change the type of phase organization in a narrow concentration range and, as a consequence, the performance properties of the final material. The sizes of phase domains in binary reactive systems are governed by their initial compatibility, the polymerization reaction kinetics, and the diffusion mobility of the components [10,11,12]. Despite numerous studies, the influence of photoinitiation conditions and thermoplastic modifier content on the kinetics of phase structure formation for specific dimethacrylate systems remains insufficiently studied.

An optical interferometry-based technique was previously developed to determine the concentration ranges for the formation of all known phase morphology types of cured thermosetting systems modified with thermoplastics [13,14]. This work aims to investigate how the evolution of phase equilibria and the macromolecular mobility of components influence the prediction of the phase structure and its parameters in photocurable dimethacrylate systems, where the reaction kinetics are significantly higher than in epoxy counterparts.

Bisphenol-A 10-ethoxylate dimethacrylate (BPADMA), commonly utilized in photocurable compositions, was selected as the dimethacrylate matrix. Polypropylene glycol (PPG) is a polyether widely employed as a plasticizer. Due to its compatibility with a number of dimethacrylate oligomers, low viscosity, and ability to alter the mobility of reaction mixture components, PPG holds significant interest as a thermoplastic modifier. The introduction of PPG can influence phase separation kinetics, material morphology, and mechanical properties [15,16]. PPG is also used as a plasticizer to improve the ductility and processability of brittle polymers [17], such as polylactide [18].

This work aims to establish the underlying principles governing phase structure formation in a PPG-modified photocurable dimethacrylate system as a function of the reaction conditions and the modifier content.

## 2. Materials and Methods

### 2.1. Materials

The studied objects were photocurable compositions based on the dimethacrylate oligomer bisphenol-A 10-ethoxylate dimethacrylate (BPADMA), containing polypropylene glycol (PPG) and a photoinitiator system consisting of camphorquinone (CQ) and 2-ethylhexyl-4-(dimethylamino)benzoate (ODMAB). Curing of the compositions was carried out using an Ekkel LED dental lamp (wavelength range of 420–480 nm, radiation intensity of 1200–1500 mW/cm^2^, Guilin Woodpecker Medical Instrument Co., Guilin, China). The structural formulas of BPADMA, PPG, CQ, and ODMAB and their manufacturers are presented in Table 1.

### 2.2. Methods

#### 2.2.1. Optical Interferometry (OI)

Mass transfer in binary gradient systems was studied using optical interferometry on an ODA-2 (IPCE RAS, Moscow, Russia). The method is based on in situ recording of the optical density distribution in the interface region between components and its change over time. A detailed diagram of the optical interferometer is presented in [13,19].

The experiment consisted of clamping one component between the glass panes of a diffusion cell coated with a semitransparent Ni-Cr alloy, positioned at an angle of 2° to form an interference picture. The second component was introduced into the gap between the glass panes (120 μm), and the interdiffusion of the components was observed.

Photocuring of the system inside the diffusion cell was carried out using an LED lamp with 2-s pulses at 1-min intervals. The interval between pulses provided sufficient time to observe and record interference pictures showing the mass transfer processes and phase separation induced by the photocuring reaction. To reduce the light intensity, the lamp was positioned 15 mm from the diffusion cell. A constant distance was maintained using a light-tight cylindrical spacer, the inner diameter of which matched the diameter of the LED lamp’s light guide.

#### 2.2.2. Scanning Electron Microscopy (SEM)

The phase structure of the cured samples was examined using scanning electron microscopy on a JSM-6060A microscope (JEOL, Tokyo, Japan). SEM images of the sample were obtained using secondary electrons at an accelerating voltage of 15 kV. The cured BPADMA+CQ/ODMAB–PPG samples were mechanically cleaved, and a platinum layer was deposited on the cleaved surface using magnetron sputtering on a DSR1 system (Nano-Structured Coatings Co., Tehran, Iran) for 20 s (by the magnetron method in argon at a current of 100 mA) to form a uniform conductive coating.

#### 2.2.3. Fourier Transform Infrared Spectroscopy (FTIR)

Conversion was determined using Fourier transform infrared spectroscopy on a Nicolet iN10 IR microscope (Thermo Scientific, Waltham, MA, USA) in the spectral range of 675–4000 cm^−1^.

The systems were studied before and after curing (either using an LED lamp for 40 s at a distance of 15 mm from the sample, or under natural daylight for 2 days) using a germanium crystal in ATR mode by accumulating 128 scans with a resolution of 4 cm^−1^ at a temperature of 20 °C, followed by processing using Omnic 9 software (Thermo Scientific, Waltham, MA, USA).

The conversion degree was calculated using the standard method (Equation (1)) widely used for acrylate dental materials [20], based on the decrease in the intensity of the absorption band at 1638 cm^−1^, corresponding to the stretching vibrations of the C=C double bond of the methacrylate group, relative to the band at 1608 cm^−1^, caused by the stretching vibrations of the aromatic ring, the intensity of which does not change during the polymerization. The intensity of the bands was estimated relative to the baseline in the range from 1660 to 1590 cm^−1^.

(1) $$\alpha =\left(1-\frac{{\left(\frac{I_{1638}}{I_{1608}}\right)}^{polymer}}{{\left(\frac{I_{1638}}{I_{1608}}\right)}^{monomer}}\right)\times 100\%$$

where ${\left(\frac{I_{1638}}{I_{1608}}\right)}^{monomer}$ is the absorption intensity of the corresponding monomer band (before curing), and ${\left(\frac{I_{1638}}{I_{1608}}\right)}^{polymer}$ is the absorption intensity of the corresponding polymer band (after curing). The FTIR spectrum of the initial PPG showed a complete absence of absorption bands in the 1700–1500 cm^−1^ range. Accordingly, the standard method for assessing the degree of acrylate group conversion using the 1638 cm^−1^ band (assigned to the 1608 cm^−1^ band) is valid.

FTIR spectra were recorded for three different samples, after which the obtained data on the conversion degree were averaged.

#### 2.2.4. Compression Tests

To evaluate the deformation of BPADMA+CQ/ODMAB–PPG samples with a PPG content of 1—0; 2—0.22; 3—0.28; 4—0.32; 5—0.52; and 6—0.72 volume fractions (vol.fr.), radial compression tests were performed in accordance with ISO 604:2002 [21] on a Z010 universal tensile testing machine (Zwick/Roell, Ulm, Germany) using a 2.5 kN force cell. The test temperature was 20 °C.

Cylindrical tablets with a diameter of 6 ± 0.01 mm and a thickness of 2 ± 0.01 mm, obtained by photocuring the reaction mixture with an LED lamp for 40 s, were used as samples. The tablets were placed on their ends and loaded until failure (maximum force dropped by 80%). The stress–strain curve was recorded and the failure mode was analyzed. For each composition, at least five independent measurements (n ≥ 5) were performed; the results are presented as mean values and standard deviations.

#### 2.2.5. Sample Preparation

To study photocuring using optical interferometry, a mixture of BPADMA and the CQ/ODMAB photoinitiator system was prepared in advance. The CQ:ODMAB weight ratio was 1:2, with the total photoinitiator content being 2% by weight relative to the BPADMA weight. The composition was prepared in a light-tight container to prevent premature polymerization. To achieve a uniform composition, the mixture was stirred at 37 °C at 1000 rpm until completely homogenized. The resulting reaction mixture was introduced into the gap between the glass surfaces of the diffusion cell, followed by contact with PPG.

To obtain samples for FTIR and SEM studies, PPG was added to the pre-prepared BPADMA+CQ/ODMAB composition in amounts of 0.22, 0.28, and 0.32 vol.fr. After the modifier was added, the compositions were mixed until a homogeneous reaction mixture was obtained.

For SEM morphology studies, the reaction mixture was placed in a cylindrical silicone mold with a diameter of 6 mm and a height of 2 mm. The samples were cured either using an LED lamp for 40 s at a distance of 15 mm from the sample (set by a distance controller) or under natural daylight for 2 days. Using both curing modes allowed us to evaluate the effect of polymerization rate on the size of the resulting phase structures. It should be noted that under natural light, the intensity and spectral composition of the radiation were not controlled and could vary depending on environmental conditions. To improve reproducibility, it is advisable to use lower-intensity LED irradiation in the future, allowing for controlled simulation of slower curing under fixed radiation parameters. Samples cured with the LED lamp were subjected to compression tests.

To determine the conversion degree, FTIR spectroscopy was used to record the spectrum from the surface of the reaction mixture placed in a silicone mold. After curing the sample for 40 s, an LED lamp was used to record the IR spectrum of the low-temperature cleavage surface, characterizing the molecular structure of the bulk material.

## 3. Results and Discussion

### 3.1. Study of Component Compatibility in the BPADMA–PPG System

Analysis of the initial state of the system, specifically assessing the thermodynamic compatibility of the components and determining the interdiffusion coefficients, is an important step before studying photocuring and structure formation processes in the BPADMA–PPG system. The initial phase state and molecular mobility of the components in the system before the onset of the chemical curing reaction are fundamental for predicting and accurately interpreting the structure formation processes and curing kinetics in the studied system.

**Figure 1:**
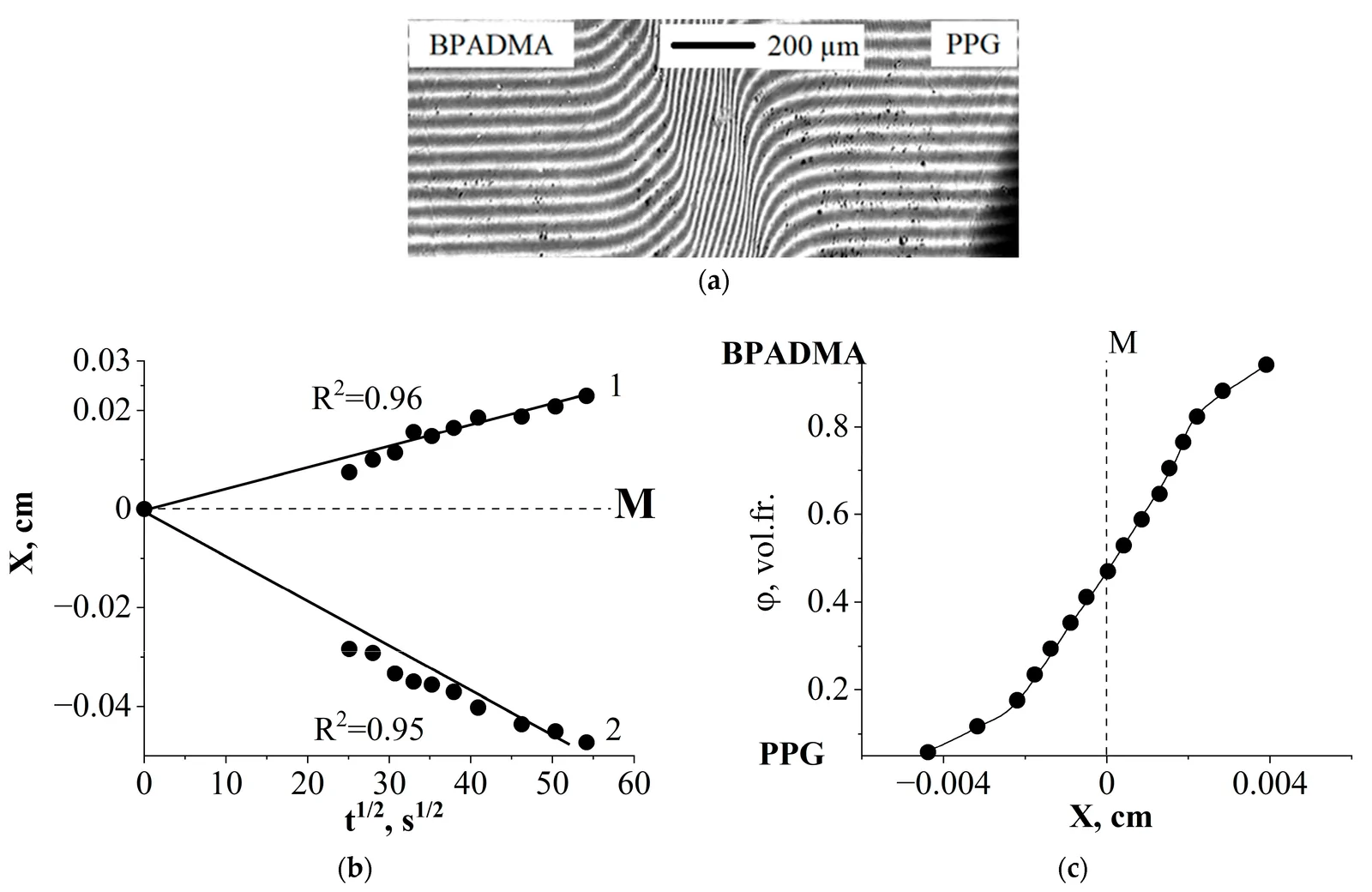
(**a**) Interferogram of the interdiffusion zone of the BPADMA–PPG system (t = 70 min, T = 20 °C). (**b**) Kinetic dependences of the movements of isoconcentration planes in the BPADMA–PPG system (1—diffusion of PPG into BPADMA, 2—diffusion of BPADMA into PPG). M is the Matano–Boltzmann plane. (**c**) Concentration distribution of components in the interdiffusion zone of the BPADMA–PPG system at 20 °C and a time of 70 min.

The compatibility of the initial components BPADMA and PPG was studied using optical interferometry at 20 °C (Figure 1a). It can be seen that all interference lines in the interdiffusion zone are optically resolved and are described by a continuous S-shaped concentration distribution profile from one component to the other (Figure 1c), indicating complete mutual solubility of the system’s components. The position of the Matano–Boltzmann plane is constant and corresponds to the mid-range of the compositions. The kinetic dependences of the isoconcentration planes characterizing the movement of BPADMA macromolecules into the PPG matrix and the movement of PPG molecules into the BPADMA matrix are linear (Figure 1b), indicating the absence of chemical interaction between the components during mixing. The mixing process is spontaneous and follows a diffusion mechanism (X ≈ √t).

Based on the kinetic dependencies of the movement of isoconcentration planes, the limiting coefficients of interdiffusion were calculated (Equation (2)), which amounted to D_v_^BPADMA^ = 10^−8^ cm^2^/s and D_v_^PPG^ = 10^−7^ cm^2^/s [22,23].

(2) $$D_{v}=\frac{1}{2}{\left(\frac{\Delta X}{\surd t}\right)}^{2}$$

### 3.2. Study of Photocuring of BPADMA+CQ/ODMAB–PPG System

In dimethacrylate–thermoplastic systems, the chemical reaction of photocuring can lead to the formation of a heterogeneous structure, which is caused by the deterioration of the system’s thermodynamic stability due to the increase in the molecular weight of dimethacrylate. Optical interferometry enables us to visually track this transition of the system from a homogeneous to a heterogeneous state.

**Figure 2:**
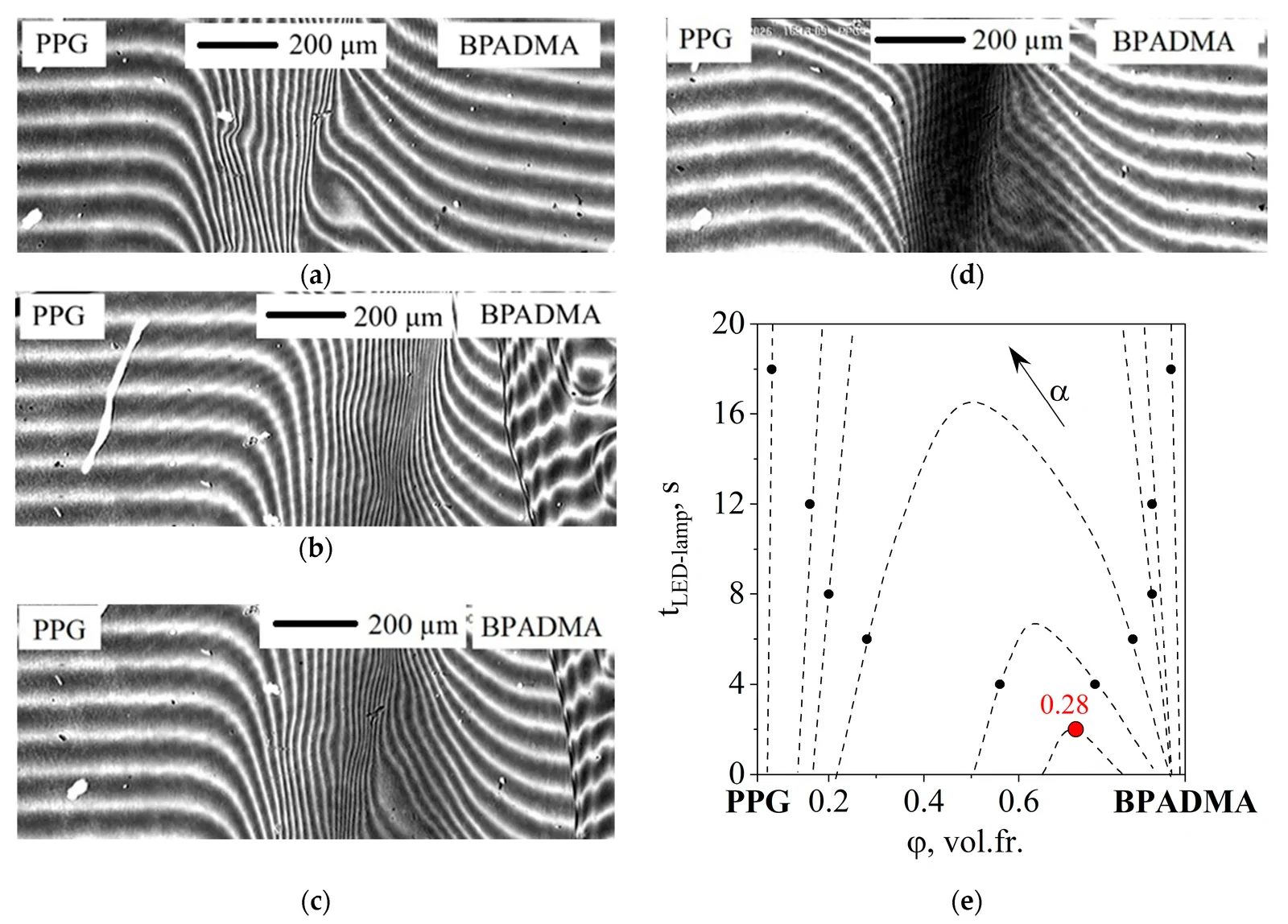
Interferograms of the BPADMA+CQ/ODMAB–PPG system during curing with an LED lamp at a distance of 15 mm with a curing time of (**a**) 0, (**b**) 2, (**c**) 6, (**d**) 18 s; and (**e**) kinetic dependence of the change in the compositions of coexisting phases (●) in the isothermal mode during the photocuring reaction (the arrow shows the movement of the model phase diagram (dotted lines)); ●—critical concentration point at the intersection of the curing isotherm with the binodal curve, determined by optical interferometry).

Thus, in the interferograms of the diffusion zone of the conjugated components PPG and BPADMA+CQ/ODMAB at the initial stage in the absence of a photocuring reaction, a continuous concentration profile is observed (Figure 2a), characterizing the system as unlimitedly compatible over the entire concentration range.

Photocuring was performed using an LED lamp with 2-s pulses at 1-min intervals. After 2 s of photocuring, a phase boundary (PB) appears in the interdiffusion zone of the components on the interferogram (Figure 2b), indicating the onset of phase separation. The concentration in the PB localization region in the interdiffusion zone should correspond to the critical concentration of the binodal dome. Thus, the position of the PB on the interferogram determines the concentration of the mixture, the curing of which leads to its placement directly in the middle of the phase reversal region of the binodal curve of the phase diagram. Therefore, the state points of the systems located in the phase reversal region should be characterized by an IPP-type structure after curing.

The photocuring process is accompanied by a broadening of the heterogeneous region in the interferogram (Figure 2c,d). The compositions of the coexisting phases, plotted on the temperature–concentration field of the phase diagram (Figure 2e), were determined from the PB width in the interferogram. With a photocuring time of 2 s under isothermal conditions (20 °C), the calculated PPG concentration in the BPADMA+CQ/ODMAB–PPG mixture, corresponding to the phase inversion region (the system composition characterized by the IPP-type structure), was 0.28 vol.fr. To the left and right of the calculated PPG concentration are the concentration ranges of the forming matrix-dispersion structures with particles enriched in one or the other component. After 18 s of photocuring, the positions of the compositions of the coexisting phases virtually cease to change, indicating the completion of the phase separation processes.

**Figure 3:**
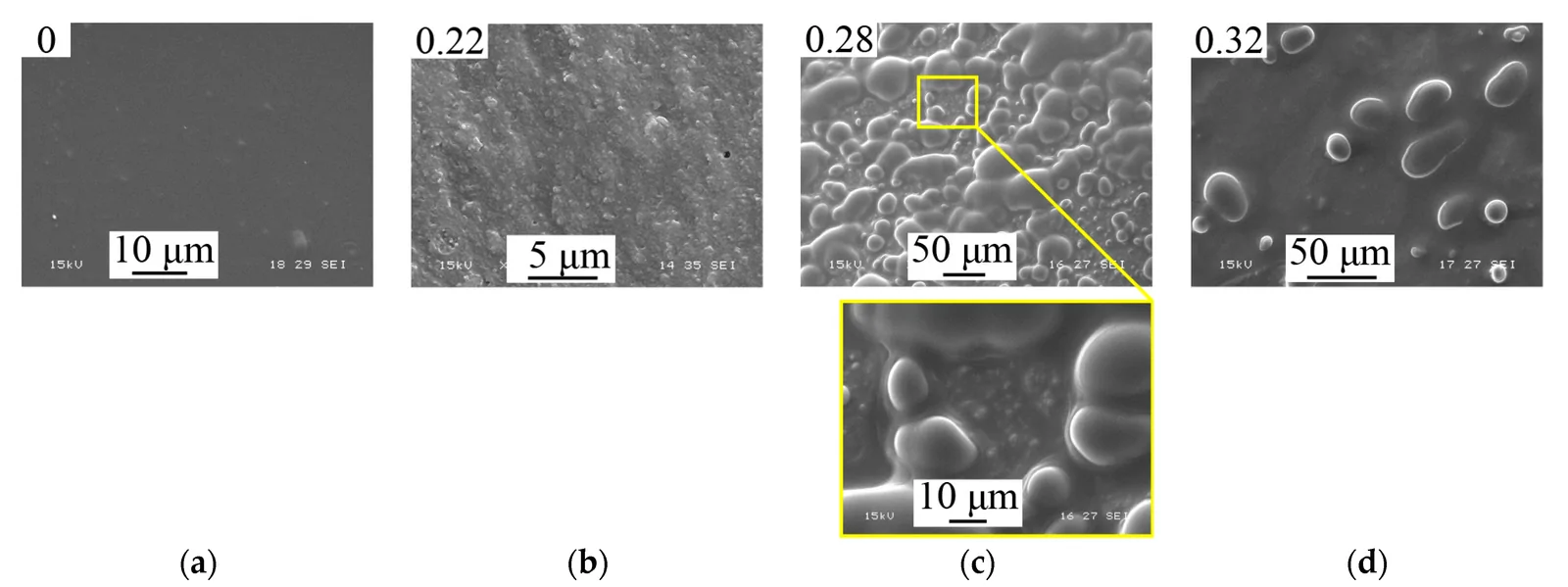
SEM images of the cured BPADMA+CQ/ODMAB–PPG system with PPG content of (**a**) 0; (**b**) 0.22; (**c**) 0.28; and (**d**) 0.32 vol.fr. under LED lamp (40 s, 15 mm distance).

Based on the optical interferometry results, BPADMA+CQ/ODMAB–PPG compositions (cured with an LED lamp at a distance of 15 mm with a curing time of 40 s) with PPG contents of 0, 0.22, 0.28, and 0.32 vol.fr. were selected. Samples were prepared for these compositions, and the morphology of the low-temperature cleavage surfaces was studied using SEM. The cured BPADMA+CQ/ODMAB sample in the absence of a modifier is characterized by a homogeneous phase structure (Figure 3a). At a PPG content of 0.22 vol.fr. in the mixture, the samples are characterized by a matrix-dispersion phase structure (Figure 3b), in which the continuous phase is enriched in BPADMA, and the dispersed phase is enriched in PPG with an average phase size of ~0.32 µm. The PPG concentration of 0.28 vol.fr. is in the concentration range of 0.27–0.30, determined using optical interferometry, and is characterized by an IPP-type structure according to SEM data (Figure 3c). For this morphology, the quantitative characteristic of the structure is the volume fraction of each of the continuous phases, which was 67:33 (extended phase enriched in PPG: extended phase enriched in BPADMA). This ratio of extended phases indicates a slight shift in the critical concentration during curing and is comparable to the error in determining the critical concentration using optical interferometry. A further increase in the concentration to 0.32 vol.fr. of PPG leads to the formation of an inverted matrix-dispersion phase structure with an average phase size of ~25 µm (Figure 3d).

Thus, the obtained results confirm the possibility of predicting the type of forming structure based on optical interferometry data.

### 3.3. Study of the Influence of Photocuring Conditions and PPG Content on the Structure and Degree of Conversion of the BPADMA+CQ/ODMAB–PPG System

The kinetics of the photocuring reaction determine the rate at which the gel point is reached and the final degree of conversion of acrylate groups. The rate of formation of the spatial network of chemical bonds significantly influences phase separation processes: a decrease in the time to reach the gel point limits the diffusion mobility of the components, which affects the size of the forming phase structures. Furthermore, the introduction of the PPG modifier dilutes the reaction medium, leading to increased mobility of reactive groups, and may affect the final conversion degree of the system. Therefore, the influence of photocuring conditions and PPG content on the phase structure and the conversion degree of the BPADMA+CQ/ODMAB–PPG system is studied.

**Figure 4:**
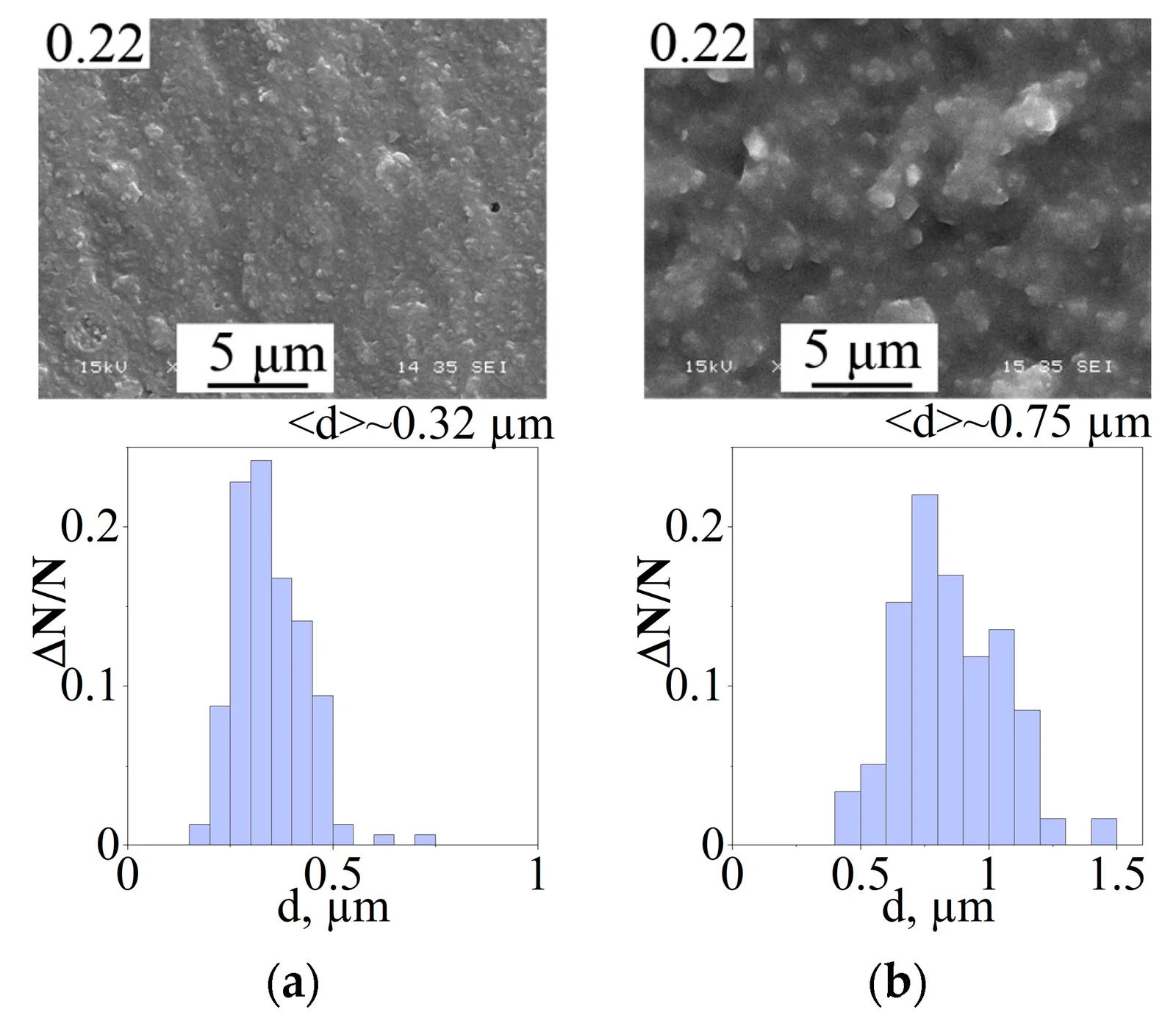
SEM images of the BPADMA+CQ/ODMAB–PPG system with a PPG content of 0.22 vol.fr. cured: (**a**) with an LED lamp (40 s, at a distance of 15 mm); (**b**) natural light (2 days). For both curing systems, the phase size distribution is given.

To evaluate the effect of curing conditions on the sizes of the formed phase structures, the morphology of BPADMA+CQ/ODMAB–PPG samples with a PPG content of 0.22 vol.fr., cured in different modes, was studied using SEM: under the influence of an LED lamp (for 40 s, at a distance of 15 mm) and during curing under natural light for 2 days. It was found that, regardless of the curing mode, in both cases the samples are characterized by a matrix-dispersion phase structure type, in which the matrix is enriched in BPADMA, and the dispersed phase is enriched in PPG (Figure 4).

When cured under natural light, the polymerization reaction rate is slower than under LED light [24]. Slower reaction kinetics increase the time it takes to reach the gel point. Moreover, phase structure formation occurs under conditions of high diffusion mobility, which facilitates the growth of the PPG-enriched dispersed phase and the formation of a large phase dispersion with an average size of ~0.75 µm (Figure 4b).

The higher LED lamp intensity in the CQ absorption region increases the polymerization rate and promotes earlier gelation. Consequently, the diffusion mobility of the components is limited early in the structure formation process, leading to structure fixation during the initial phase formation stages. As a result, significantly finer dispersed phases are formed compared to samples cured under natural light (average size ~0.32 µm) (Figure 4a).

**Figure 5:**
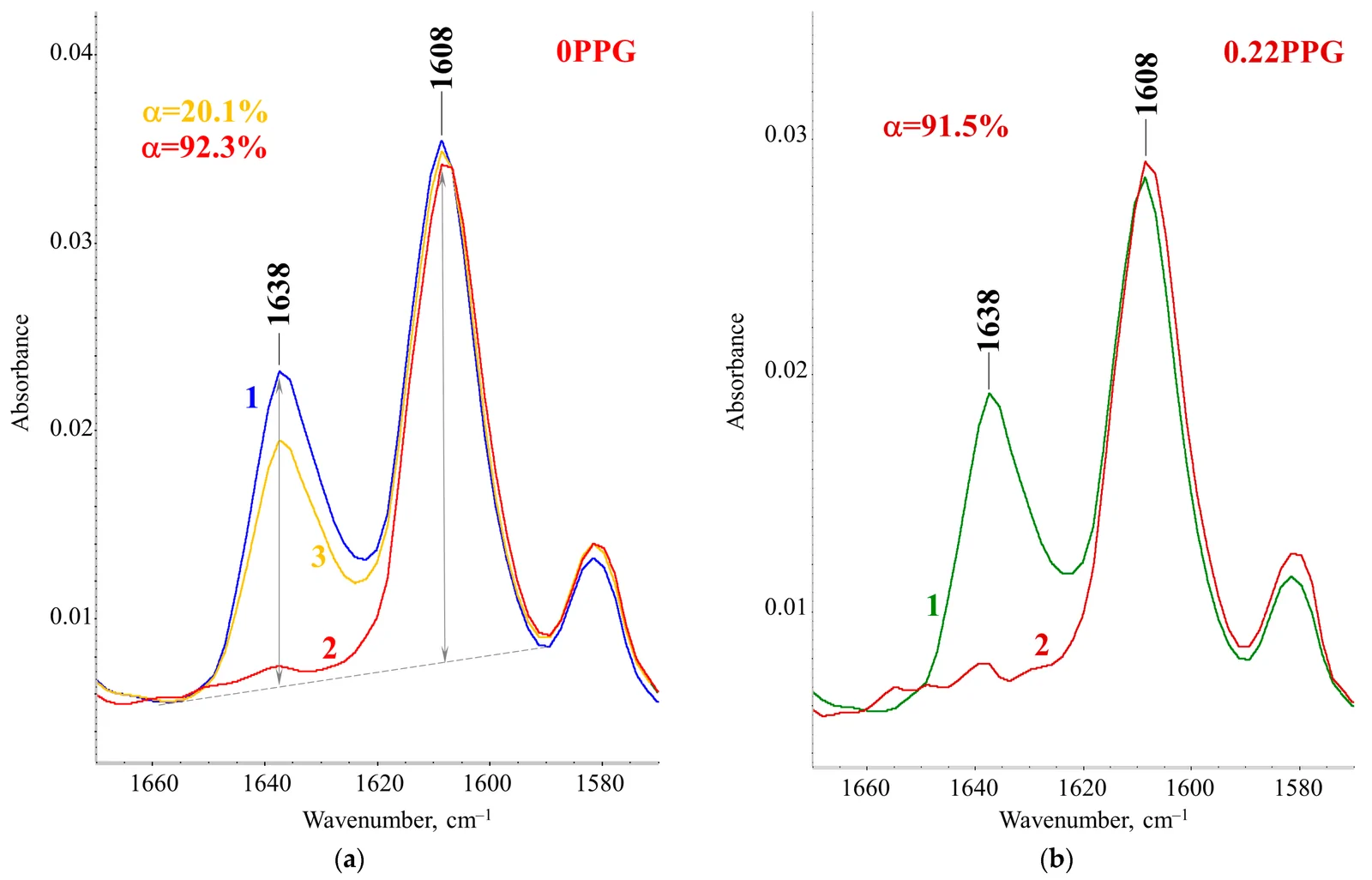
FTIR spectra of systems (**a**) BPADMA+CQ/ODMAB and (**b**) BPADMA+CQ/ODMAB–PPG with a PPG content of 0.22 vol.fr. The curves correspond to: 1—uncured system; 2—spectrum of a cleavage sample (characterizing the volume of material) cured with an LED lamp (40 s); 3—spectrum from the surface of a sample cured with an LED lamp (40 s).

FTIR spectroscopy revealed (Figure 5) that conversion degree values depend significantly on the analysis region of the cured sample. When recording an IR spectrum from the surface of a sample cured with an LED lamp, the average conversion of acrylate groups was 20.1 ± 4.78% (yellow line in Figure 5a). Meanwhile, analysis of a cleavage sample of the same sample, characterizing the bulk of the material, revealed a conversion of approximately 92.3 ± 2.85% (red line in Figure 5a). This difference is due to the inhibition of polymerization as a result of the oxidative action of atmospheric oxygen in contact with the surface layers of the system [25]. Inhibition leads to an increase in the number of unreacted methacrylate groups in the surface layer of the material. Within the bulk of the sample, the influence of oxygen is limited, so the photocuring process proceeds to a higher conversion degree of the functional groups.

A study of the effect of PPG (0.22 vol.%) on the degree of material conversion based on IR spectra from the cleaved surface of the samples showed that the introduction of the modifier has virtually no effect on the final conversion degree. For the BPADMA+CQ/ODMAB system, the average conversion degree was 92.3 ± 2.85%, while for the BPADMA+CQ/ODMAB–PPG (0.22 vol.%) system it was 91.5 ± 2.73% (Figure 5b). However, the difference between the samples cured with an LED lamp and in natural light is more noticeable: 85.1 ± 0.81% for the BPADMA+CQ/ODMAB system and 86.8 ± 1.90% for the BPADMA+CQ/ODMAB–PPG system with a PPG content of 0.22 vol.fr. This correlates well with the SEM data and is due to the lower reaction rate under natural light conditions. Consequently, the influence of the modifier is manifested mainly in the phase structure and, probably, in the molecular structure of the polymer network.

### 3.4. Study of the Effect of PPG Content on the Elasticity of the BPADMA+CQ/ODMAB–PPG System

**Figure 6:**
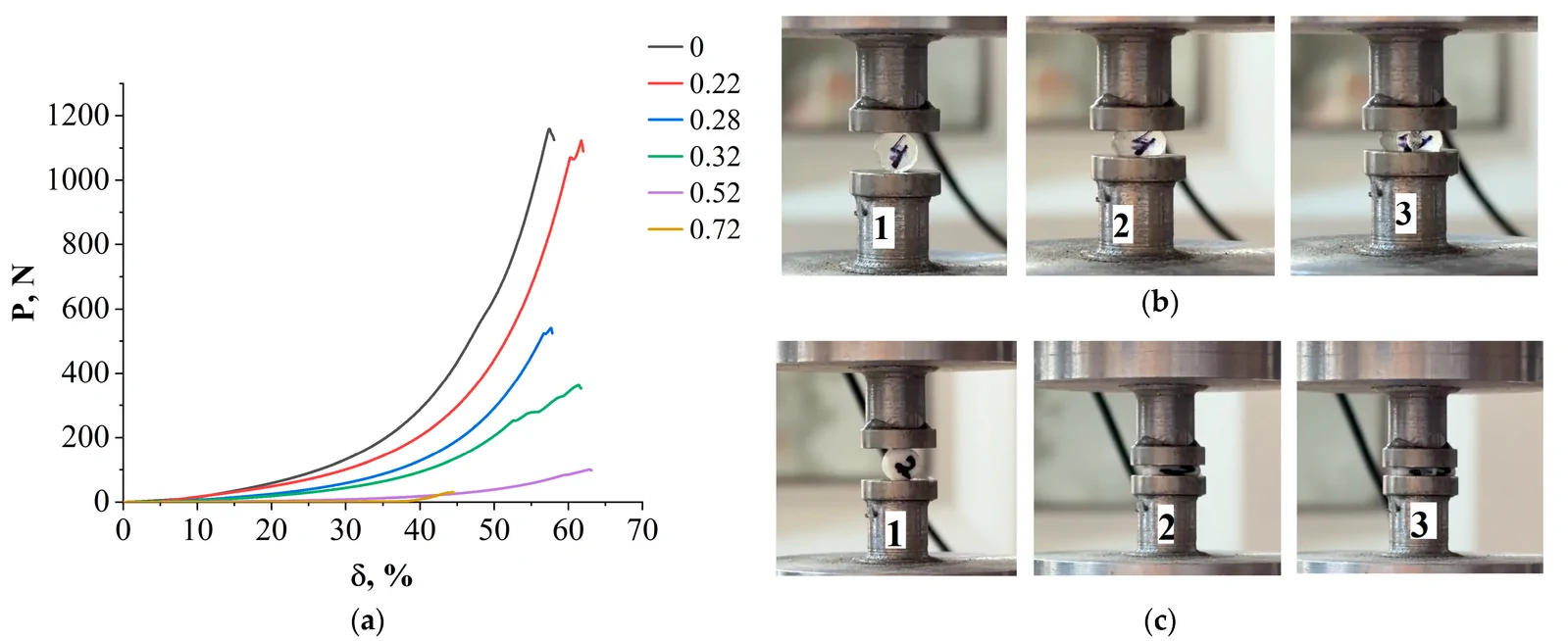
(**a**) Stress–strain curves during compression testing of LED-cured BPADMA+CQ/ODMAB–PPG. The legend indicates the PPG content as a vol.fr. Photos during testing of BPADMA+CQ/ODMAB–PPG samples containing PPG: (**b**) 0; (**c**) 0.70 vol.fr., where 1—sample before testing; 2—maximum compression of sample before failure; 3—failure of sample.

Modifying the BPADMA+CQ/ODMAB system with PPG resulted in significant elasticization of the material. To assess the effect of PPG concentration on material deformation, mechanical radial compression tests were conducted on LED-cured samples. They exhibited plastic deformation under loading across the entire studied concentration range. As the PPG content in the system increased, the nature of the stress–strain curves changed (Figure 6). With increasing PPG content in the system, the compressive failure force changes (Figure 6, Table 2). The unmodified BPADMA+CQ/ODMAB system exhibits the highest compressive strength compared to the modified system, owing to the formation of a homogeneous polymer network. The presence of long ethoxylated fragments in the BPADMA structure ensures flexibility of the polymer chains, enabling the material to deform without fracture. The BPADMA+CQ/ODMAB–PPG system, with a PPG content of 0.22 vol.fr., exhibits similar compressive strength values, owing to the matrix-dispersion phase structure, in which the continuous phase is the cross-linked BPADMA network, while PPG is present as a dispersed phase and has no significant effect on the material’s deformability.

For a sample with a PPG content of 0.28 vol.fr, the phase structure is characterized by an IPP type, which leads to greater deformation capacity of the material due to a reduced volume fraction of the continuous phase enriched in BPADMA, which provides deformation resistance. The second extended phase, enriched in PPG, is characterized by increased deformability.

When switching to an inverted structure, in which the continuous phase is enriched with PPG, a decrease in compressive strength and an increase in material elasticity are observed. The most pronounced elasticity effect is observed at a PPG content of 0.72 vol.fr. in the BPADMA+CQ/ODMAB–PPG system. This effect may be due not only to changes in the phase structure but also to the plasticizing effect of PPG. Dilution of BPADMA with PPG modifier leads to a decrease in crosslink density, which contributes to a decrease in stiffness and deformation resistance. Thus, the observed decrease in compressive strength is determined by the combined effect of changes in the phase structure of the material and the plasticizing effect of PPG, which is accompanied by a decrease in the effective crosslink density.

## 4. Conclusions

Photopolymerization-induced phase separation in a polypropylene glycol-modified dimethacrylate system was studied. Optical interferometry revealed that the initial system is thermodynamically compatible, but that photopolymerization results in decreased component compatibility due to increased molecular weight and the formation of a spatially cross-linked dimethacrylate network, leading to phase separation.

The applicability of a previously developed optical interferometry-based technique for determining the concentration point corresponding to the center of the phase inversion region (PPG 0.28 vol.fr.) was confirmed. It was established that the PPG content influences the formation of the desired phase structure, while the polymerization kinetics influence the size of the final phase structures. The results indicate that the introduction of non-reactive polypropylene glycol enables targeted control of the morphology of the photocurable dimethacrylate system.

The established regularities are of practical interest for developing photopolymer materials with controlled mechanical properties, including materials with increased flexibility.

## Figures and Tables

**Table 1:** Initial components, and their structural formulas and manufacturers.

Name	Structure	Manufacturer
BPADMA Bisphenol-A 10-ethoxylate dimethacrylate (M_w_ = 808 g/mol)	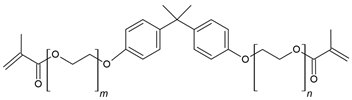	Alfa Chemical, Zhengzhou, China CAS:41637-38-1
PPG Polypropylene glycol (M_w_ = 2000 g/mol)	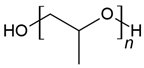	Alfa Chemical, Zhengzhou, China CAS: 25322-69-4
CQ Camphorquinone	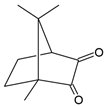	Alfa Chemical, Zhengzhou, China CAS: 10373-78-1
ODMAB 2-Ethylhexyl-4-(dimethylamino)benzoate	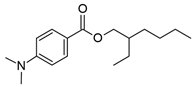	Alfa Chemical, Zhengzhou, China CAS: 21245-02-3

**Table 2:** Radial compression failure stress values for BPADMA+CQ/ODMAB–PPG specimens.

φ_PPG_, vol.fr.	Strength, MPa	φ_PPG_, vol.fr.	Strength, MPa
0	44.72 ± 4.15	0.32	12.76 ± 1.25
0.22	40.41 ± 4.33	0.52	3.72 ± 0.31
0.28	17.38 ± 1.36	0.72	1.21 ± 0.12

## Data Availability

Data are available upon request to the authors.
